# The bacteria of Yangtze finless porpoise (*Neophocaena asiaeorientalis asiaeorientalis*) are site-specific and distinct from freshwater environment

**DOI:** 10.3389/fmicb.2022.1006251

**Published:** 2022-12-20

**Authors:** Xizhao Zhang, Congping Ying, Min Jiang, Danqing Lin, Lei You, Denghua Yin, Jialu Zhang, Kai Liu, Pao Xu

**Affiliations:** ^1^Key Laboratory of Freshwater Fisheries and Germplasm Resources Utilization, Ministry of Agriculture and Rural Affairs, Freshwater Fisheries Research Center, Chinese Academy of Fishery Sciences, Wuxi, China; ^2^Wuxi Fisheries College, Nanjing Agricultural University, Wuxi, China

**Keywords:** Yangtze finless porpoise, site-specific, freshwater environment, bacteria, bacterial community, bacterial function

## Abstract

Bacteria play an essential role in the health of marine mammals, and the bacteria of marine mammals are widely concerned, but less is known about freshwater mammals. In this study, we investigated the bacteria of various body sites of Yangtze finless porpoise (*Neophocaena asiaeorientalis asiaeorientalis*) and analyzed their association with freshwater environmental bacteria. The bacterial community and function of Yangtze finless porpoise showed apparent site-specificity. Various body sites have distinct differences in bacteria and have their dominant bacteria. *Romboutsia*, *Plesiomonas*, *Actinobacillus*, *Candidatus Arthromitus* dominated in the intestine (fecal and rectal samples). *Fusobacterium*, *Streptococcus*, and *Acinetobacter* dominated in the oral. The dominant genera in the blowhole include *Suttonella*, *Psychrobacter*, and two uncultured genera. *Psychrobacter*, *Flavobacterium*, and *Acinetobacter* were dominant in the skin. The alpha diversity of intestinal (fecal and rectal) bacteria was the lowest, while that of skin was the highest. The oral and skin bacteria of Yangtze finless porpoise significantly differed between the natural and semi-natural conditions, but no sex difference was observed. A clear boundary was found between the animal and the freshwater environmental bacteria. Even the skin bacteria, which are more affected by the environment, are significantly different from the environmental bacteria and harbor indigenous bacteria. Our results provide a comprehensive preliminary exploration of the bacteria of Yangtze finless porpoise and its association with bacteria in the freshwater environment.

## Introduction

Microbiota broadly affects the growth and development of aquatic mammals, playing a role in nutrition, immunity, health, etc (Sanders et al., 2015; Mootapally et al., 2017; Di Guardo et al., 2019). In the more extensive study of marine mammals, researchers describe in detail the microbial composition and structure of different body sites of various marine mammals. Body sites, feeding habits, habitats, and phylogeny are the main factors affecting the microbial communities of marine mammals (Nelson et al., 2015; Sanders et al., 2015; Bik et al., 2016; Apprill et al., 2020). The study of marine mammalian microbiota provides valuable insights into the health status of marine mammals. In contrast to marine mammals, research on the diversity and composition of freshwater mammalian microbiota is relatively limited. The lack of microbial knowledge of freshwater mammals is detrimental to the health monitoring and conservation activities of freshwater mammals.

Yangtze finless porpoise (*Neophocaena asiaeorientalis asiaeorientalis*, YFP) inhabits the Yangtze River basin for life, is the only freshwater porpoise in the world and is endangered (Turvey et al., 2007; Mei et al., 2014; Huang et al., 2020). YFP occupies the top ecological niche of the Yangtze River ecosystem, is carnivorous, and is a unique species for studying freshwater mammals (Huang et al., 2017; Zhou et al., 2018). Currently, the studies related to the bacteria of YFP mainly focus on the fecal bacteria, mainly in the capture and semi-natural environments (McLaughlin et al., 2011, 2012, 2013; Wan et al., 2016b; You et al., 2020). In addition, *Aeromonas veronii* associated with the YFP disease has also been reported (Liu et al., 2018, 2020). Other body sites have been studied primarily in culture-based studies of respiratory tract microbiota (Wan et al., 2016a), with fewer reports about skin and oral bacteria. The lack of research on the microbiota of YFP limits our understanding of the health condition of YFP.

Environmental microbiota is one of the driving forces to shape marine mammalian bacteria, and it is also a potential source of marine mammal bacteria (Bik et al., 2016; Liu et al., 2018, 2020; Tian et al., 2022). Related studies have shown that environmental bacteria can colonize marine mammals and impact marine mammals’ health (Raverty et al., 2017; Huggins et al., 2020). Although there is a close relationship between environmental bacteria and marine mammalian bacteria, the boundary between them is also apparent (Apprill et al., 2014; Bik et al., 2016; Chiarello et al., 2017; You et al., 2020). Even the skin bacteria in close contact with the water environment differ from the environmental bacteria (Chiarello et al., 2017; Apprill et al., 2020). This association between marine mammals and environmental bacteria has been proven in many marine mammals. However, the association between freshwater mammalian and environmental bacteria needs further demonstrated.

This study collected the intestinal (rectal and fecal), blowhole, oral, and skin samples from 15 YFPs, including animal-related freshwater environmental samples. We analyzed the bacterial diversity and composition of different body sites of YFP and characterized the specific bacteria of different body sites. Differences in the YFP bacteria between the natural and semi-natural conditions were assessed. The functional specificity of the YFP bacteria was then analyzed. Finally, the association between YFP and freshwater environmental bacteria was investigated. These results provide an increased understanding of the diversity and composition of YFP bacteria, explore the characteristics of YFP bacterial communities and the influence of the freshwater environment on them, and offer a new reference for health monitoring and population conservation of YFP.

## Materials and methods

### Sample collection

The subjects included 15 YPFs from the Anqing section of the Yangtze River (natural) and the *ex-situ* conservation base of YFP in Xijiang, Anqing City (semi-natural). The animals were obtained in the same method as described in the previous studies (Yin et al., 2021). All the YFPs involved in the study were not treated with antibiotics or other drugs before sampling. The sampling process follows the general practice of veterinary nursing of marine mammals (Dierauf and Gulland, 2001). Sampling was carried out after the YFP was lifted from the water and placed on a soft sponge pad. All sterile cotton swabs were moistened with sterile saline, the gingival sulcus of the mandible was wiped with sterile cotton swabs to obtain oral samples, and the forehead skin of YFP was wiped with sterile cotton swabs to obtain skin samples. Each sample contains three swabs. The blowhole samples were collected by non-invasive method, and the 50 ml sterile polypropylene centrifuge tube was wetted with sterile saline, which was placed 10 cm above the blowhole, and the samples were collected after three breaths. The Fecal and rectal samples were collected through a sterile plastic tube with a diameter of 4 mm, and the front end was lubricated with Vaseline. Insert the fecal tube into the rectum 20 cm deep, take it out after 30 s, cut the tube into segments, collect the part containing feces as fecal samples, and collect the plastic tube in contact with the rectum (that is, the end of the insertion part of the tube) as rectal samples. Freshwater samples were collected with a 50 ml sterile polypropylene centrifuge tube in the animal activity area and filtered with fiberglass filter paper with a diameter of 47 mm and pore diameter of 0.22um. The filter paper is stored in a 1.5 ml sterile centrifuge tube containing alcohol. In addition, there are two stool samples and two water environment samples from the previous study (You et al., 2020). Detailed sample collection information can be found in Supplementary Table S1.

### DNA extraction, amplification, and sequencing

The sample is transferred to a new 2 ml aseptic centrifuge tube, and the filter paper is cut into pieces before transfer. An appropriate amount of guanidine thiocyanate and N-lauroyl sarcosine were added to each tube at 70°C and transferred to a tissue lytic apparatus for oscillation after 1 h. After centrifuging the sample, the supernatant was removed and transferred to the 2 ml centrifuge tube. 500 μL temp was added to the residual sample, stirred, swirled, and centrifuged. Use the pipette to absorb 500 μL of supernatant and repeat the previous step 3 times. Once again, the supernatant was centrifuged at high speed, transferred to two isopropanol tubes, and stored overnight at 4°C. The sample was centrifuged at high speed, and the supernatant was discarded in next day. Phosphate buffer and potassium acetate are added to one sample sedimentation tube, transferred to another tube, and mixed. It was placed on ice for 1.5 h and centrifuged at 4°C for 30 min. Add anhydrous ethanol and NaAc and let it stand at −20°C for several hours. After centrifugation, the supernatant was removed, and 70% ethanol was added to separate the washing solution by centrifugation. After repeated washing and adding TE solution, DNA was obtained after dissolving. Remove samples with low DNA quality (total mass < 1.0 μg, concentration < 10 ng/μL, moderately or severely degraded). The V3-V4 region of 16SrDNA was amplified by primers 341F (ACTCCTACGGGAGGCAGCAG) and 806R (GGACTACHVGGGTWTCTAAT). The conditions were pre-denaturation at 94°C for 3 min, followed by 30 cycles of denaturation at 94°C for 30s, annealing at 55°C for 30s, extension at 72°C for 30s, and then at 72°C for 5 min to complete the reaction. It was excised from agarose gel and purified by the Universal DNA purification kit (Tiangen, China). The purified product was subjected to high-throughput sequencing on the Illumina Miseq 2000 platform (BGI, Shenzhen, China).

### Data analysis

First, the primers and barcodes were removed from the raw data of high-throughput sequencing. Microbiome bioinformatics analysis was performed using QIIME 22022.2 (Bolyen et al., 2019). Raw sequence data were demultiplexed and quality filtered using the q2-demux plugin, then denoising using DADA2 (Callahan et al., 2016). SILVA 13 8 99% reference sequences (Quast et al., 2013) were assigned to amplicon sequence variants (ASVs) using the classify-sklearn naïve Bayesian classifier (McDonald et al., 2012) of the q2 feature classifier (Bokulich et al., 2018). Removal of non-bacterial ASVs and chloroplast-associated ASVs. All ASVs were aligned to mafft (Katoh et al., 2002) and used to construct a phylogeny tree using fasttree2 (Price et al., 2010). Alpha diversity metrics (ACE, Chao1, Shannon, and Faith’s phylogenetic diversity) and beta diversity metric (Bray Curtis dissimilarity) were calculated after all samples were sparsed (subsampling without replacement) to the lowest sequences number of all samples. Non-metric multidimensional scaling (NMDS) and Permutational multivariate analysis of variance (PERMANOVA) are all implemented with the Vegan v2.5–7 package (Oksanen et al., 2020). The unweighted pair-group method with arithmetic means (UPGMA) clustering by ggtree v3.6.1 package (Yu et al., 2017). All heatmaps are drawn with ComplexHeatmap v2.3.1 package (Gu et al., 2016). Shared genera were created by the UpSetR v1.4.0 package (Conway et al., 2017). Source track analysis was performed by SourceTrack2 (Knights et al., 2011). The LefSef analysis (Segata et al., 2011) performed *via* Galaxy (Afgan et al., 2018). Bacterial function was analyzed by PICRUSt2 (Douglas et al., 2020). All visualizations in this study were implemented with R v4.1.1 (R Core Team, 2021).

## Results

### Bacterial composition of Yangzte finless porpoise

**Figure 1:**
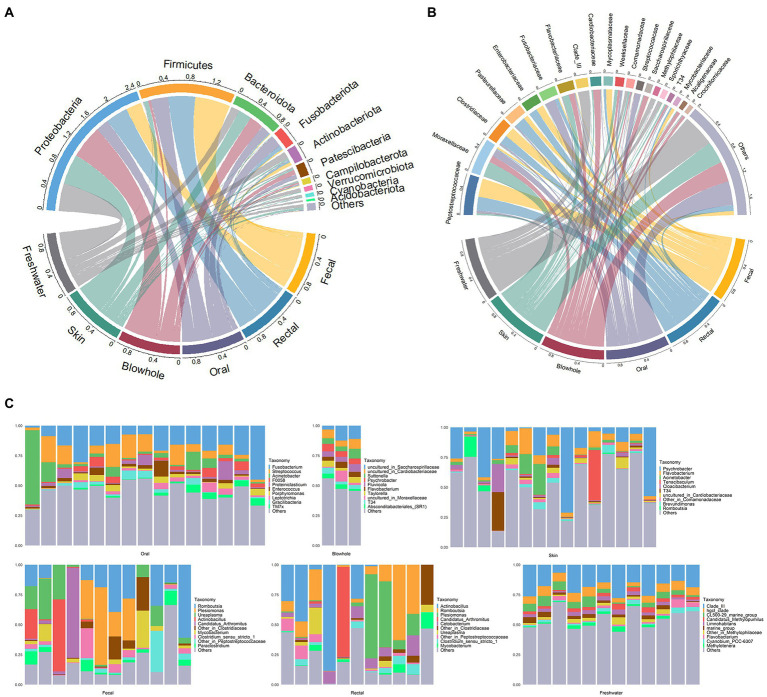
Bacterial composition of YFP and the freshwater environment. **(A)** The circos plot of bacterial composition at the phylum level. The top 10 phyla with an average scale are displayed. **(B)** The circos plot of bacterial composition at the family level. The top 20 families with an average scale are displayed. **(C)** The bar plot of bacterial composition at the genus level. The top 10 genera with an average scale are displayed in each sample.

The four phyla, Proteobacteria, Firmicutes, Bacteroidota, and Fusobacteriota, were dominant in YFP (Figure 1A). Moreover, Actinobacteria were more abundant in the intestinal samples (fecal: 6.0 ± 2.9%, rectal: 3.5 ± 1.4%, Mean ± Standard Error). Patescibacteria was dominant in the oral (7.6 ± 1.6%) and blowhole samples (8.3 ± 1.7%, Figure 1A). The dominant bacteria in the fecal and rectal samples at the family level were Peptostreptococcaceae (fecal: 32 ± 7.3%, rectal: 24 ± 6.7%), Clostridiaceae (fecal: 17 ± 6.2%, rectal: 15 ± 6.7%), and Enterobacteriaceae (fecal: 13 ± 6.1%, rectal: 14 ± 7.5%, Figure 1B). In addition, Mycoplasmataceae (10 ± 3.7%) was abundant in the fecal samples, and Pasteurellaceae (18 ± 8.6%) was abundant in the rectal samples (Figure 1B). The bacteria of oral samples were dominated by Fusobacteriaceae (17 ± 2.7%), Moraxellaceae (13 ± 4.1%), and Streptococcaceae (9.9 ± 1.9%, Figure 1B). Cardiobacteriaceae (13 ± 2.3%), Moraxellaceae (11 ± 3%), Flavobacteriaceae (9.2 ± 0.7%), and Saccharospirillaceae (8.9 ± 2.9%) were abundant in the blowhole samples (Figure 1B). In the skin samples, Moraxellaceae (26 ± 5.3%), Flavobacteriaceae (11 ± 3.6%), and Weeksellaceae (5.5 ± 2%) were dominant bacteria at the family level (Figure 1B).

At the genus level, *Romboutsia* (fecal: 20 ± 5.3%, rectal: 16 ± 6%), *Plesiomonas* (fecal: 13 ± 6.1%, rectal: 14 ± 7.5%), *Actinobacillus* (fecal: 7.3 ± 5.2%, rectal: 18 ± 8.6%), *Candidatus Arthromitus* (fecal: 6.8 ± 6.2%, rectal: 7 ± 6.8%) dominated in the fecal and rectal samples (Figure 1C). *Ureaplasma* (9.9 ± 3.5%) in the fecal samples and *Cetobacterium* (5.6 ± 1.6%) in the rectal samples were abundant (Figure 1C). The oral bacteria was dominated by *Fusobacterium* (17 ± 2.7%), *Streptococcus* (9.8 ± 1.9%), and *Acinetobacter* (8.7 ± 4.0%, Figure 1C). The dominant genera in blowhole include *Suttonella* (5.5 ± 1.3%), *Psychrobacter* (5.2 ± 0.52%), and two uncultured genera assigned to Saccharospirillaceae (8.9 ± 2.9%) and Cardiobacteriaceae (6.9 ± 0.98%, Figure 1C). *Psychrobacter* was dominant in the skin samples, accounting for nearly 20% (20 ± 5.6%). Other dominant genera in the skin samples were *Flavobacterium* (6.9 ± 1.4%) and *Acinetobacter* (4.7 ± 1.9%, Figure 1C).

### Bacterial diversity of Yangtze finless porpoise

**Figure 2:**
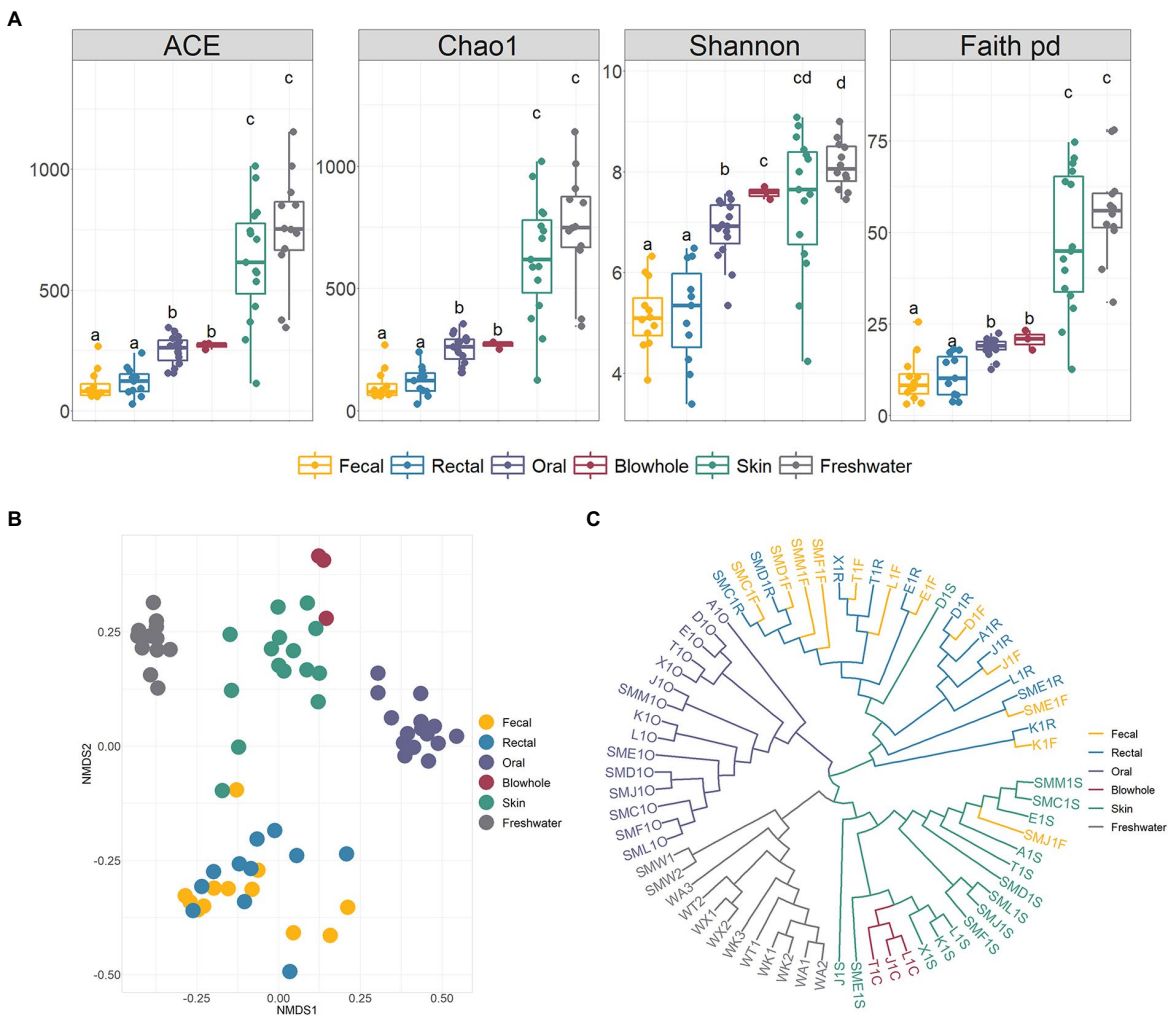
Alpha and beta diversity of bacteria in the YFP and the freshwater. **(A)** Alpha diversity (ACE, Chao1, Shannon, Faith PD). Wilcoxon test calculates statistical significance between samples, with different letters indicating significant differences (*p* < 0.05). **(B)** NMDS ordination based on Bray Curtis dissimilarity across various body sites and the freshwater. **(C)** The unweighted pair-group method with arithmetic means (UPGMA) clustering analysis base on Bray Curtis dissimilarity. The meaning of the sample corresponding to the label is shown in Supplementary Table S1.

Shannon rarefaction curves showed sufficient coverage of diversity in all samples (Supplementary Figure S1). The bacterial alpha diversity metrics (ACE, Chao1, Shannon, Faith PD) between various body sites of YFP showed that the skin had the highest diversity, significantly higher than other body sites. On the contrary, the intestinal bacteria (fecal and rectal) was the lowest alpha diversity (Figure 2A). No significant alpha diversity difference was found between fecal and rectal samples (Wilcoxon test, *p* > 0.05, Figure 2A). There were no significant differences in alpha diversity between oral and blowhole samples, except for the Shannon metric.

The NMDS ordination, UPGMA clustering, and PERMANOVA analysis based on Bray Curtis dissimilarity demonstrated significant divergence of bacteria in various body sites (PERMANOVA, *p* < 0.01, Figures 2B,C; Supplementary Table S2). The intestinal, oral, blowhole and skin bacteria showed an evident dissimilarity to each other and constructed four distinct clusters (PERMANOVA, *p* < 0.001, Figures 2B,C; Supplementary Table S2). In particular, no significant differences between the fecal and rectal samples (PERMANOVA, *p* > 0.05, Figure 2B), and the fecal and rectal samples were gathered in the same cluster (Figure 2C).

### Bacteria in different conditions

**Figure 3:**
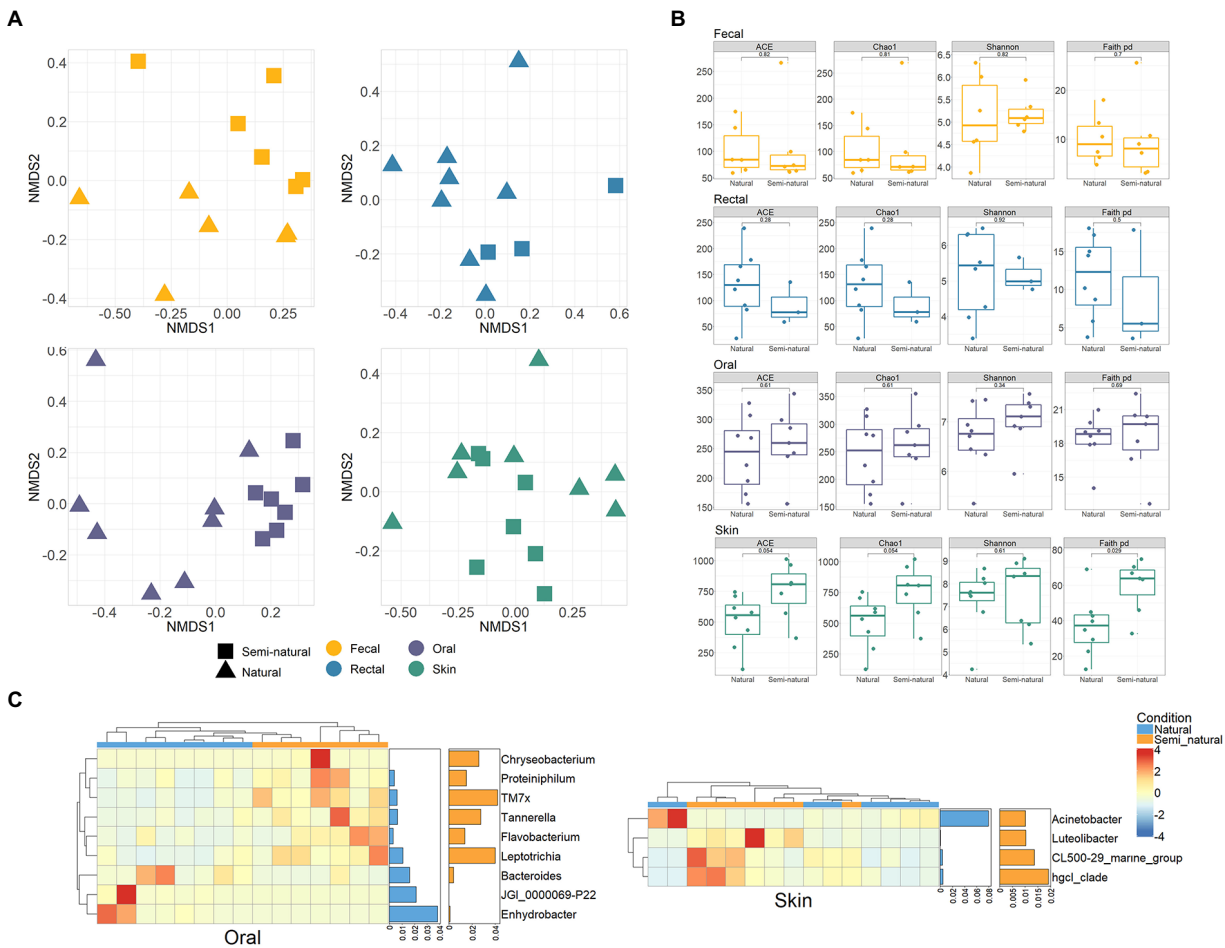
Bacterial differences in the natural and semi-natural condition. **(A)** NMDS ordination based on Bray Curtis dissimilarity between the natural and semi-natural condition in four body sites of YFP. **(B)** Comparison of alpha diversity differences of YFP bacteria under two conditions. Wilcoxon test calculates statistical significance between samples. **(C)** Heatmap of the oral and skin differential bacteria (genus level) between the natural and semi-natural conditions. Each row and column of the heatmap corresponds to genus and samples, respectively. The row data for each genus were z-score transformed. The bar plot on the right represents the average proportion of bacteria in two conditions. Wilcoxon test was used to test the significance (*p* < 0.05), and the genera whose proportion was less than 1% were excluded.

Compare the bacterial differences of YFP in different sexes and conditions. The results showed no differences in the bacterial communities between sexes (PERMANOVA, *p* > 0.05, Supplementary Table S2). The YFP in natural and semi-natural conditions also did not show significant community differences in alpha and beta diversity of the intestinal (fecal and rectal) bacteria (PERMANOVA, *p* > 0.05, Supplementary Table S2 and Supplementary Figures S3A,B). However, the skin and oral bacteria were different between the two conditions. The Faith PD metric of skin bacteria in the semi-natural is significantly more than that in the natural condition (Wilcoxon test, *p* < 0.05, Figure 3B). A significant difference exists between the oral bacterial communities of the YFP in natural and semi-natural conditions (PERMANOVA, oral: *p* < 0.001, skin: *p* < 0.05, Figure 3A; Supplementary Table S2). *Enhydrobacter*, *JGI 0000069-P22*, and *Bacteroides* were significantly abundant in the oral bacteria of the YFP in natural conditions. In contrast, *Chryseobacterium*, *Proteiniphilum*, *TM7x*, *Tannerella*, and *Flavobacterium* were abundant in the semi-natural YFP (Wilcoxon test, *p* < 0.05, Figure 3C). Four significantly different genera of skin bacteria were found. *Acinetobacter* was abundant in the oral of the natural YFP, and *Luteolibacter*, *CL500-29 marine group*, *hgcl clade* were higher in the oral of the semi-natural YFP (Wilcoxon test, *p* < 0.05, Figure 3C). By comparing the bacterial differences in the freshwater environments between different conditions, 18 differential bacteria were found, of which 14 were abundant in the semi-natural conditions (Supplementary Figure S2).

### Bacterial function of Yangtze finless porpoise

**Figure 4:**
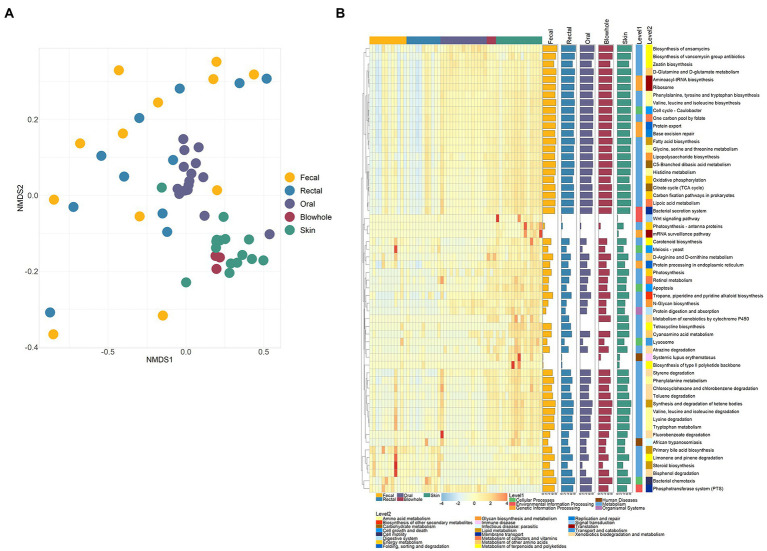
Bacterial functions of various body sites. **(A)** NMDS ordination of PICRUSt2 predicted functions using KEGG orthology (KO) based on Bray Curtis dissimilarity. **(B)** Heatmap of significant pathways in various body sites. Each row and column of the heatmap corresponds to a pathway and samples, respectively. The row data for each pathway were z-score transformed. The bar plot on the right represents the average abundance of the pathway (data is logarithmically converted). Kruskal–Wallis test was used to calculate the significance and correction by Bonferroni (*p* < 0.05).

The NMDS ordination and PERMANOVA analysis show significant differences in bacterial functions in different body sites of YFP (PERMANOVA, *p* < 0.001, Figure 4A; Supplementary Table S3). In particular, the function of fecal and rectal bacteria did not differ significantly, nor did the function of blowhole and skin bacteria (PERMANOVA, *p* > 0.05, Figure 4A; Supplementary Table S3). The bacterial function of each body site showed no significant difference in different sexes and conditions (PERMANOVA, *p* > 0.05, Supplementary Table S3).

The KO results were further inferred to the KEGG pathway level, and 58 pathways with significant differences were identified. Of these pathways, 41 pathways are categorized as Metabolism, five as Cellular Processes, and six as Genetic Information Processing (Figure 4B). The intestinal bacteria were significantly more abundant in Limonene and pinene degradation, Bisphenol degradation, Tetracycline biosynthesis, Carotenoid biosynthesis, Steroid biosynthesis than in other sites (Figure 4B). The six pathways, D-Glutamine and D-glutamate metabolism, Aminoacyl-tRNA biosynthesis, Biosynthesis of vancomycin group antibiotics, Ribosome, Phosphotransferase system (PTS), and Zeatin biosynthesis, were abundant in the oral bacteria (Figure 4B). The blowhole and skin bacterial function were abundant in Biosynthesis of ansamycins, D-Glutamine and D-glutamate metabolism, Phenylalanine, tyrosine and tryptophan biosynthesis, Tropane, piperidine and pyridine alkaloid biosynthesis, etc (Figure 4B).

### Freshwater environmental bacteria

**Figure 5:**
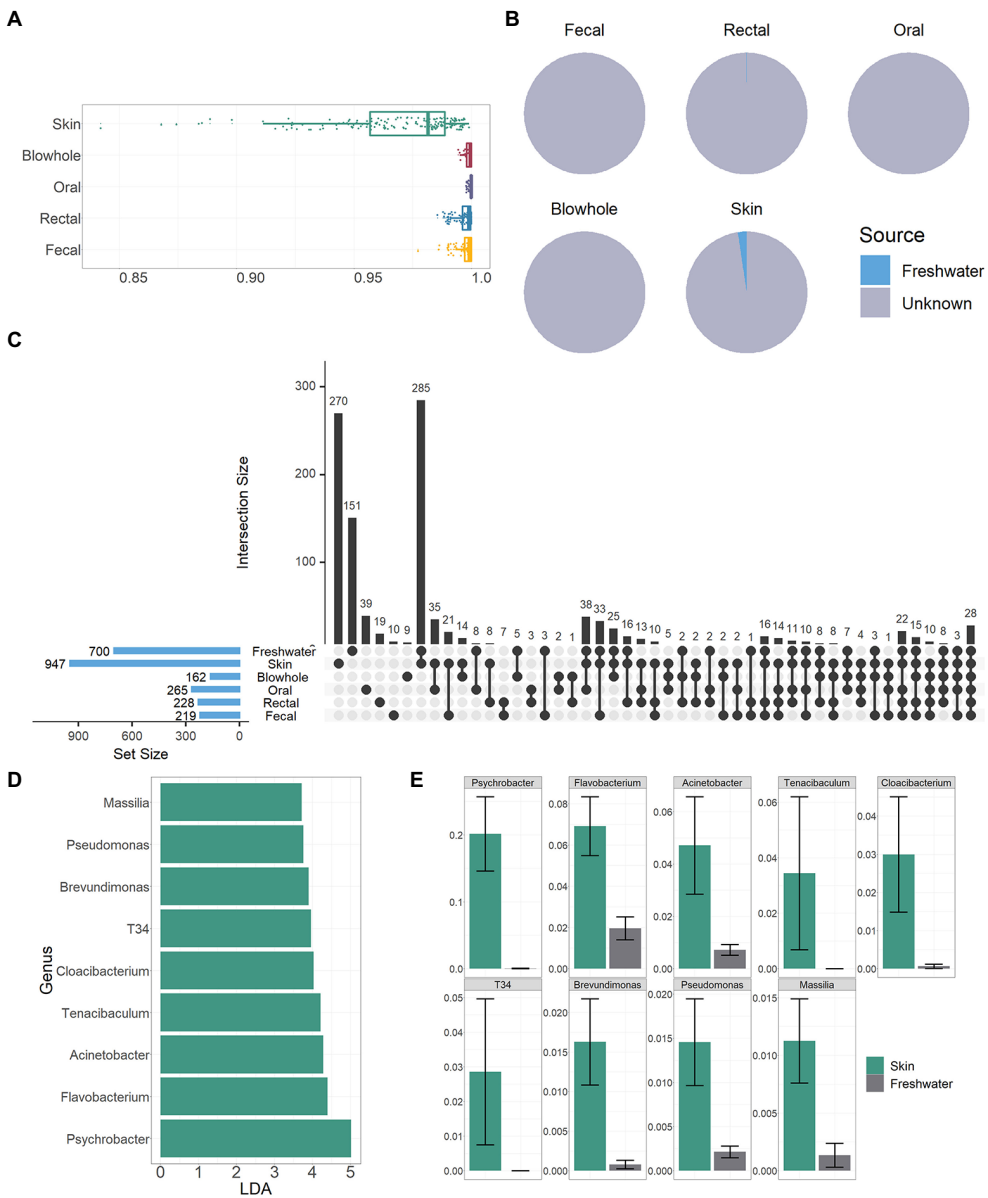
Association of freshwater environmental bacteria with YFP. **(A)** Bray Curtis dissimilarity between the freshwater environmental and YFP bacteria, the closer the value is to one, the greater the dissimilarity. **(B)** The pie chart demonstrates the source of the YFP bacteria. **(C)** Vertical bars of the upper plot show number of intersecting genera among the YFP and freshwater environments, denoted by connected black circles below the histogram. Horizontal bars show genus set size. **(D)** LDA value of nine genera. **(E)** The proportion of nine bacterial genera in the skin and freshwater environment.

The bacterial community in the freshwater environment was significantly different from that of YFP body sites (PERMANOVA, *p* < 0.01, Figure 2B; Supplementary Table S2). The dominant bacteria in the freshwater environment were *Clade III* (19 ± 1.8%), *hgcI clade* (6.5 ± 0.48%), and *CL500-29 marine group* (4.6 ± 0.79%, Figure 1C). However, the similarity between freshwater environment bacteria and skin bacteria is higher than that of other body sites (PERMANOVA, *p* > 0.05, Figure 2B, Supplementary Table S2). Bray Curtis distance between skin and freshwater is 0.97 ± 0.0021, and the mean distance between freshwater and other body sites is above 0.99 (Figure 5A). The alpha diversity of bacteria in the skin and freshwater environments did not show significant differences (Wilcoxon test, *p* > 0.05, Figure 2A). SourceTrack analysis showed that an average of 2.4% of the skin bacteria was sourced from freshwater environment, while the freshwater environmental associated bacteria in other body sites were less than 0.1% (Figure 4B). In addition, the shared bacterial genera between the skin and freshwater environments reached 285, significantly higher than other body sites (Figure 5C). The shared bacteria accounted for 30% of the total genus of skin and 41% of the total genus of the freshwater environment (Figure 5C). Considering the proximity of freshwater environmental bacteria to skin bacteria, the indigenous bacteria on the skin of YFP were further analyzed by LefSe. A total of nine genus-level bacteria significantly enriched in the skin (LDA >3, mean > 1%) were identified. *Psychrobacter* was significantly more abundant on the skin than in freshwater environments (skin: 20 ± 5.6%, freshwater: 0.045 ± 0.028%, LDA =5.0, Wilcoxon Test, *p* < 0.01, Figures 4D,E). *Flavobacterium* was also enriched on the skin (skin: 6.9 ± 1.4%, LDA = 4.4, Wilcoxon Test, *p* < 0.01, Figures 5D,E). In particular, *T34* and *Tenacibaculum* were not present in freshwater environments.

## Discussion

This study used 16 s-rRNA high-throughput sequencing technology to characterize the bacterial diversity and composition of various body sites of YFP. The bacteria of YFP show apparent body site-specificity, the boundaries of bacteria in different body sites are obvious, and each body site has its dominant bacteria. This site-specific feature is also evidenced in the bacterial function of YFP. Our results are consistent with previous studies on mammals such as humans (Lloyd-Price et al., 2016), macaques (Chen et al., 2018), dolphins, and sea lions (Bik et al., 2016), where body sites are a significant factor in constructing mammalian microbiota. Previous studies have suggested that body sites often act as environmental filters, hindering the colonization of bacteria not equipped to survive in their respective environments, allowing body sites to form distinct microbial communities (Pereira and Berry, 2017; García-Bayona and Comstock, 2018; Rojas et al., 2020). The site-specificity of YFP bacteria inspired us to consider the influence of microbiota in studying the structural characteristics and physiology of various body sites of YFP.

The intestinal bacteria of YFP was the first to attract the attention of researchers (McLaughlin et al., 2011, 2012, 2013; Wan et al., 2016b; You et al., 2020). We identified some dominant genera not characterized in previous studies, including *Romboutsia*, *Candidatus Arthromitus*, *Plesiomonas*, and *Actinobacillus*. *Romboutsia* is highly adapted to the intestinal environment and has the ability to ferment amino acids (Gerritsen, 2015; Gerritsen et al., 2019). *Plesiomonas* is a bacteria that mainly lives in various water environments (Janda et al., 2016), can cause gastrointestinal diseases in humans, and have a certain degree of antibiotic resistance (Hong et al., 2019; Cortés-Sánchez et al., 2021). Although there are no studies of this bacteria causing disease in YFP, it is worth paying attention to. *Cetobacterium* was found in the intestines of a variety of marine mammals (Bik et al., 2016; Suzuki et al., 2021; Bai et al., 2022) and can produce vitamin B12 (Suzuki et al., 2022), which may be a potential probiotic for YFP. In this study, there was no significant difference in the diversity and composition of fecal and rectal bacteria, which indicated that fecal and rectal samples were equivalent in characterizing intestinal microbiota. This result reminds us that we can choose a less invasive sampling method according to the actual situation to obtain intestinal bacteria samples of YFP.

Many studies have investigated the oral microbiota of marine mammals and discovered many novel bacteria in the oral cavity of dolphins (Bik et al., 2016; Dudek et al., 2017; Soares-Castro et al., 2019), but little known about the oral bacteria of YFP. The high abundance of *Fusobacterium* found in the oral samples of YFP is consistent with previous findings in several species of dolphins (Bik et al., 2016; Soares-Castro et al., 2019), indicating that *Fusobacterium* is a common microbe in the oral cavity of cetaceans. *Streptococcus* is considered the dominant genus in the healthy human oral cavity and plays a vital role in the assembly of the oral microbiota (Abranches et al., 2018). Characterizing the oral bacteria of YFP enhanced our understanding of the oral health status of YFP.

Skin is an essential organ of aquatic mammals and is colonized by a rich microbial community (Apprill et al., 2011, 2014; Bik et al., 2016; Chiarello et al., 2017; Li et al., 2019). The skin of YFP has the highest bacterial diversity and has unique indigenous bacteria. *Flavobacterium*, *Psychrobacter*, and *Acinetobacter* predominate in the skin of YFP. Combined with previous studies (Apprill et al., 2011, 2020), these genera may be a class of microbiota broadly associated with the skin of aquatic mammals. *Flavobacterium* often plays the role of pathogenic bacteria in freshwater aquaculture systems (Duchaud et al., 2018; Pérez-Pascual et al., 2021), but its function and pathogenicity on the skin of YFP have not been studied. In particular, The *Tenacibaculum* and *T34* were only found in the skin but not in the freshwater environment. *Tenacibaculum* is a bacteria that causes skin ulcers in fish (Olsen et al., 2022). *T34* is a class of uncultivated bacteria belonging to Burkholderiales (Kim et al., 2022). The function of these bacteria on the animal is unknown. Presently, the potential sources of *Tenacibaculum* and *T34* on the skin have not been determined, which is worthy of further analysis of their sources and functions.

Blowhole microbiota is a crucial index to evaluate the health status of aquatic mammals, and researchers collect qualified samples of blowhole microbiota through drones and other methods (Nelson et al., 2019; Centelleghe et al., 2020; Vendl et al., 2021). Considering the low extraction rate of blowhole samples in this study (only three blowhole samples were successfully extracted), we thought that perhaps the number of breaths collected should be increased at the time of sample collection to increase the amount of microbial DNA in the samples. Our findings are similar to those of a study in the blowhole of bottlenose dolphins (Lima et al., 2012), where the Saccharospirillaceae and Cardiobacteriaceae were the dominant bacteria. However, other studies on cetaceans have different results (Apprill et al., 2017; Nelson et al., 2019; Centelleghe et al., 2020), which may require further comprehensive cross-ocean mammalian blowhole microbial studies to explain. Some species in *Suttonella* can cause avian pneumonia (Peniche et al., 2017; Fischer et al., 2021), and it has not been reported that such bacteria can cause respiratory disease in YFP, but it needs attention.

Based on the body site-specific, we explored the differences between sexes and between natural and semi-natural conditions in YFP bacteria. There is no unanimous conclusion on the effect of sex on marine mammal microbiota (Apprill et al., 2014; Bik et al., 2016; Chiarello et al., 2017; Rojas et al., 2020). In this study, the operation mode of sex on the YFP bacteria was not found, indicating that bacteria differences between male and female YFP need to be further studied. This study initially showed that porpoises with different living conditions differed in oral and skin bacteria, while no differences were shown in intestinal bacteria. The differences in freshwater environmental bacteria could explain some of the reasons for the differences in skin and oral bacteria, such as *Flavobacterium* and *Luteolibacter*. The difference in microbiota in aquatic mammals under different living conditions is one of the crucial concerns in conservation biology (Bik et al., 2016; Wan et al., 2016b; Trevelline et al., 2019). Natural and semi-natural YFP may differ in the food source, water environment, and population size. While referring to previous studies of aquatic mammals (Bik et al., 2016; Wan et al., 2016b; Tian et al., 2022), these factors may be the potential factors affecting the bacteria of the YFP. Further research on the natural and semi-natural microbiota of YFP is of great significance for the *ex situ* conservation assessment of YFP.

Our study shows that the association between the YFP bacteria and freshwater environmental bacteria is consistent with previous findings on marine mammals that a clear boundary is maintained between animal and environmental bacteria (Apprill et al., 2014; Bik et al., 2016; Chiarello et al., 2017; You et al., 2020). There are apparent differences in the community structure and composition between YFP and the freshwater environment. Of course, the difference between YFP and environmental bacteria does not mean there is no mutual communication between the animal and environment. SourceTrack analysis showed that freshwater environmental bacteria were one of the sources of YFP. There are more bacteria from the freshwater environment in the skin, and very few in other parts, indicating that the freshwater environment bacteria have different effects on the bacteria of different body sites of YFP. The microbial environment background in this study only focuses on freshwater environment, and both food and air may be the source of bacteria in various body sites of aquatic mammals (Bik et al., 2016; Vendl et al., 2019). The role of YFP and the bacteria in diverse environmental contexts deserves more research to improve our insights into habitat selection and assessment of YFP from a microbial perspective.

In summary, we provide a more comprehensive bacterial map of an endangered natural animal, Yangtze finless porpoise. This study characterized the bacterial composition and diversity of the intestine (fecal and rectal), oral, blowhole, and skin of YFP. The bacteria differed significantly between different body sites and had their major bacteria, showing clear body site specificity. Meanwhile, the bacterial function of YFP also showed site-specificity. The bacterial communities of YFP in natural and semi-natural conditions were significantly different. There is a clear boundary between freshwater environmental bacteria and the YFP bacteria. The skin bacteria are relatively more affected by the freshwater environment bacteria. Our study shows the basic information on the YFP bacteria and the influence of the freshwater environment and provides a reference for the research on the YFP bacteria. Further research on the bacteria of YFP is of great significance for protecting YFP.

## Data availability statement

The raw data of this study have been submitted to the NCBI database under the project number PRJNA857500. The code in the study has been uploaded to Github (https://github.com/CesarZhang/YZFP-16s).

## Ethics statement

The animal study was reviewed and approved by the Wildlife Protection Law of the People’s Republic of China China’s Laboratory animal—Guideline for Ethical Review of Animal Welfare (GB/T 35892-2018) Office of Fisheries Law Enforcement for Yangtze River Basin of Ministry of Agriculture and Rural Affairs of the People's Republic of China (2017 [185]) and the Fisheries Bureau of Anqing City, Anhui Province, respectively.

## Author contributions

XZ wrote the original manuscript and performed the data analysis and visualization. CY, KL, and PX reviewed and edited the article. MJ, LY, DY, and JZ participated in the investigation of this study. DL provided the necessary resources for this study. All authors contributed to the article and approved the submitted version.

## Funding

This work was supported by the National Key R&D Program of China (No, 2021YFD1200304) and project of the Yangtze finless porpoise protection and implementation in the middle and lower reaches of the Yangtze River.

## Conflict of interest

The authors declare that the research was conducted in the absence of any commercial or financial relationships that could be construed as a potential conflict of interest.

## Publisher’s note

All claims expressed in this article are solely those of the authors and do not necessarily represent those of their affiliated organizations, or those of the publisher, the editors and the reviewers. Any product that may be evaluated in this article, or claim that may be made by its manufacturer, is not guaranteed or endorsed by the publisher.
