# Supplementary material for: The bacteria of Yangtze finless porpoise (Neophocaena asiaeorientalis asiaeorientalis) are site-specific and distinct from freshwater environment
Source: Front Microbiol. 2022 Dec 20;13:1006251. doi: 10.3389/fmicb.2022.1006251 (PMC9808046; doi:10.3389/fmicb.2022.1006251)
Supplement: Supplementary file 4 [file Data_Sheet_1.docx]

Supplementary Material


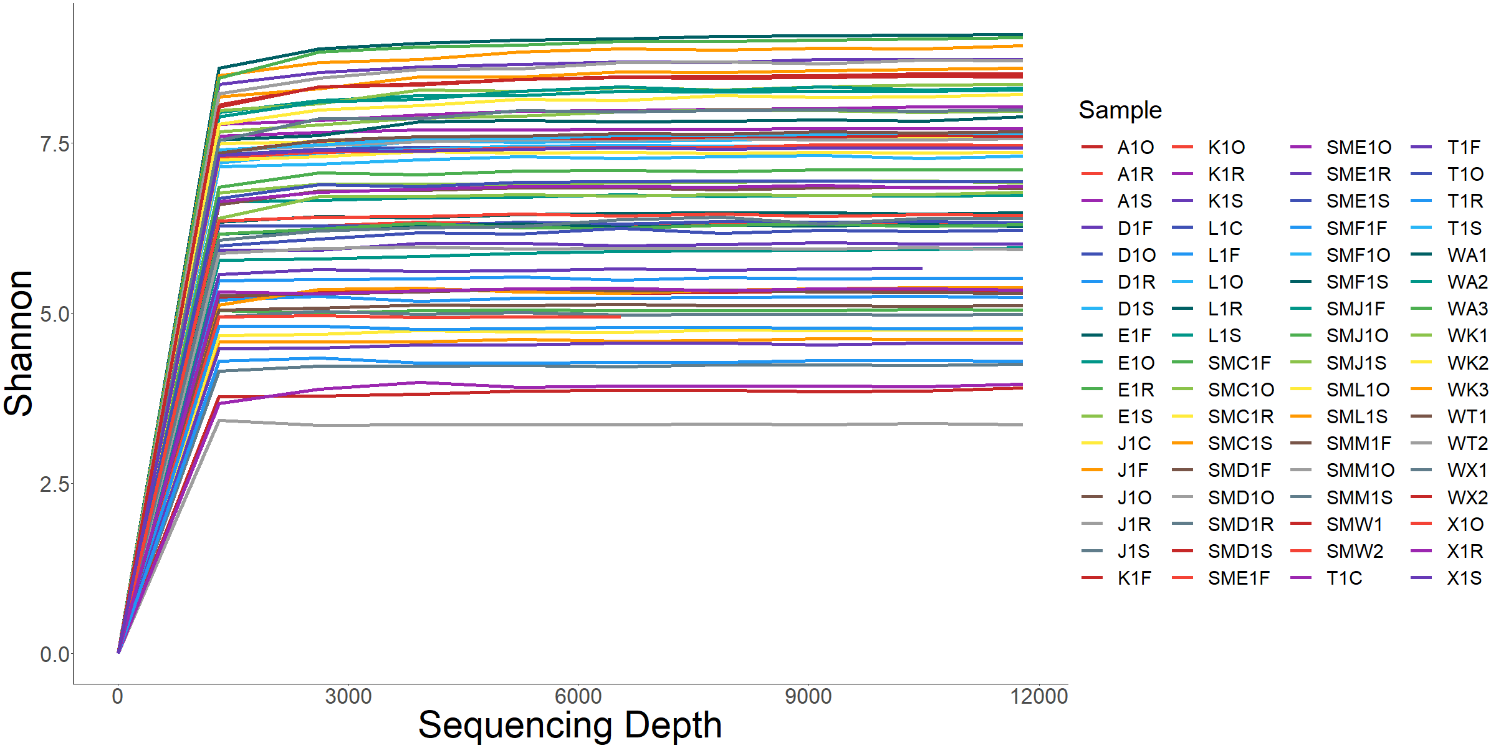


**Supplementary Figure S1.** Shannon rarefaction curves for each sample from YFP.


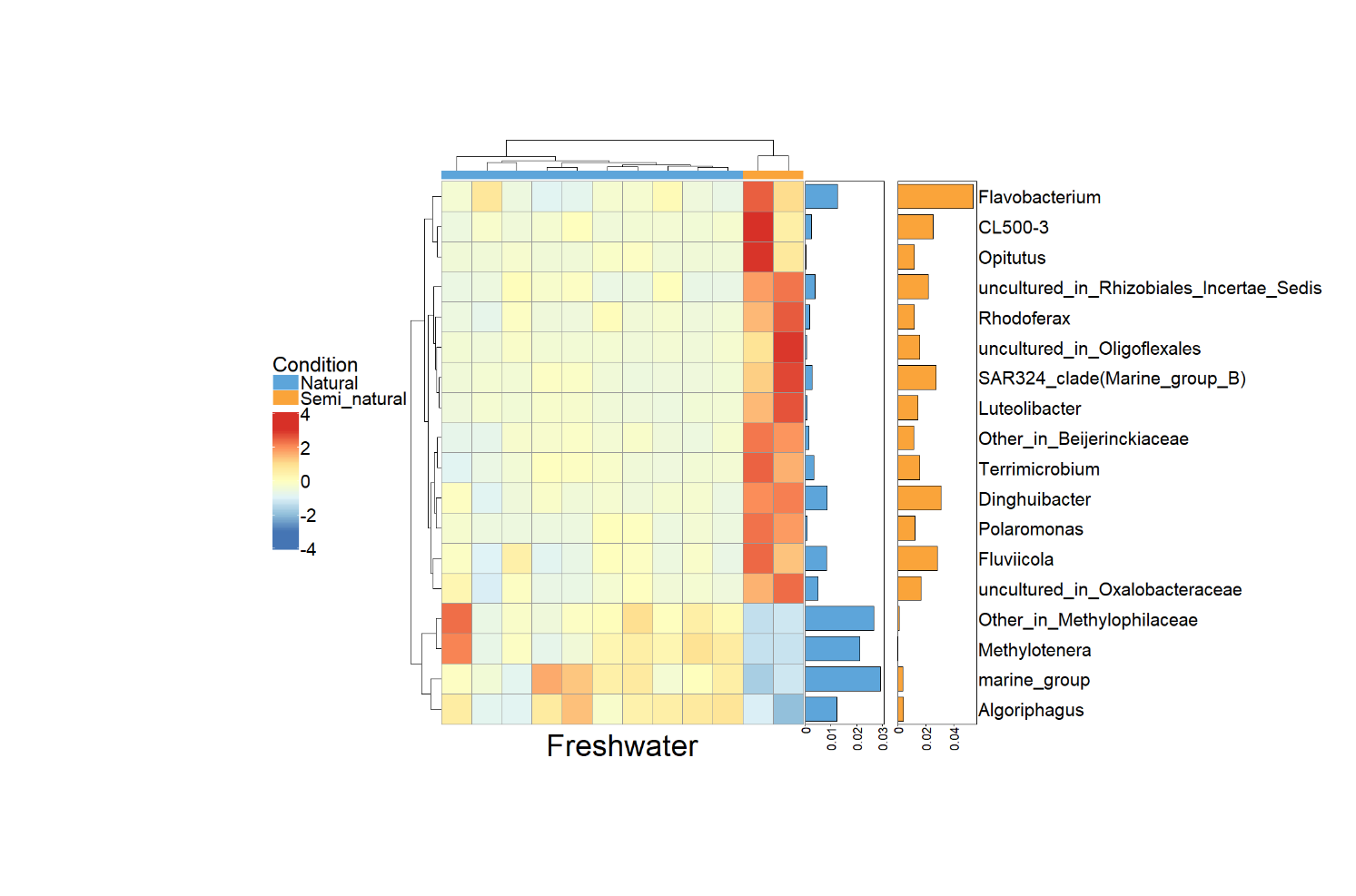


**Supplementary Figure S2.** Heatmap of differential bacteria of frreshwater environment (genus level) between natural and semi-natural conditions. Each row and column of the heatmap corresponds to genus and samples, respectively. The row data for each genus were z-score transformed. The bar plot on the right represents the average proportion of bacteria in two conditions. Wilcoxon test was used to test the significance (*P*<0.05), and the genera whose proportion was less than 1% were excluded.
